# Deep Learning Analysis of In Vivo Hyperspectral Images for Automated Intraoperative Nerve Detection

**DOI:** 10.3390/diagnostics11081508

**Published:** 2021-08-21

**Authors:** Manuel Barberio, Toby Collins, Valentin Bencteux, Richard Nkusi, Eric Felli, Massimo Giuseppe Viola, Jacques Marescaux, Alexandre Hostettler, Michele Diana

**Affiliations:** 1 Department of Research, Institute of Image-Guided Surgery, IHU-Strasbourg, 67091 Strasbourg, France; valentin.bencteux@ihu-strasbourg.eu (V.B.); eric.felli@dbmr.unibe.ch (E.F.); 2 Department of Research, Research Institute against Digestive Cancer, IRCAD, 67091 Strasbourg, France; toby.collins@ircad.fr (T.C.); jacques.marescaux@ircad.fr (J.M.); alexandre.hostettler@ircad.fr (A.H.); michele.diana@ircad.fr (M.D.); 3 Department of Surgery, Ospedale Card. G. Panico, 73039 Tricase, Italy; masgiuviola@yahoo.it; 4 Department of Research, Research Institute against Digestive Cancer, IRCAD Africa, Kigali 2 KN 30 ST, Rwanda; ricardonkusi@gmail.com; 5 ICUBE Laboratory, Photonics Instrumentation for Health, 67412 Strasbourg, France

**Keywords:** hyperspectral imaging, artificial intelligence, tissue recognition, intraoperative navigation tool, optical imaging, deep learning, precision surgery, convolutional neural network

## Abstract

Nerves are critical structures that may be difficult to recognize during surgery. Inadvertent nerve injuries can have catastrophic consequences for the patient and lead to life-long pain and a reduced quality of life. Hyperspectral imaging (HSI) is a non-invasive technique combining photography with spectroscopy, allowing non-invasive intraoperative biological tissue property quantification. We show, for the first time, that HSI combined with deep learning allows nerves and other tissue types to be automatically recognized in in vivo hyperspectral images. An animal model was used, and eight anesthetized pigs underwent neck midline incisions, exposing several structures (nerve, artery, vein, muscle, fat, skin). State-of-the-art machine learning models were trained to recognize these tissue types in HSI data. The best model was a convolutional neural network (CNN), achieving an overall average sensitivity of 0.91 and a specificity of 1.0, validated with leave-one-patient-out cross-validation. For the nerve, the CNN achieved an average sensitivity of 0.76 and a specificity of 0.99. In conclusion, HSI combined with a CNN model is suitable for in vivo nerve recognition.

## 1. Introduction

Minimally invasive surgery offers obvious advantages to patients, including a faster postoperative recovery and a shorter hospital stay. To improve surgical performances, intraoperative imaging modalities might enhance the surgical vision and assist the surgeon in the tasks of recognizing important anatomical structures that need to be safeguarded or selectively removed [1].

Specifically, nerves are fundamental structures that need to be preserved, and an inadvertent nerve injury might result in an important function loss with a consistent negative impact on the patient’s quality of life. Iatrogenic nerve injury might occur in general during surgical procedures, and precise nerve identification remains a challenging task in both open and minimally invasive surgery. In fact, despite technical improvements in intraoperative imaging systems, nerves remain difficult to visualize. In particular, after laparoscopic colorectal resections, the incidence of fecal incontinence resulting from damage to the pelvic autonomic nerves remains high, with a rate of 40–60% [2].

On the other hand, during an open surgical procedure, such as thyroidectomy, the identification of the recurrent laryngeal nerve is a key step to avoid an iatrogenic injury. However, although permanent vocal cord palsy caused by recurrent laryngeal nerve injury is rare, ranging from 0.5% to 1.5%, it still remains a considerable burden, considering that thyroidectomy is one of the most frequently performed surgical procedures worldwide [3].

The difficulty of identifying nerves intraoperatively is principally attributed to their small size and tubular shape. Furthermore, in their original anatomical positions, nerves are often embedded into a sheet of connective and fatty tissue, together with blood vessels: the neurovascular bundle. As a result, distinguishing a nerve from other tissue types, such as small venous or arterial blood vessels, surrounding fatty tissue intraoperatively can be rather challenging.

An intraoperative guidance tool able to precisely and objectively discriminate and identify the nerves would provide important assistance to the surgeon to prevent iatrogenic injuries during open and laparoscopic operations. In this view, hyperspectral imaging (HSI) represents a promising technology.

HSI, an optical imaging technology, combining a photo camera with a spectrometer, provides both spatial and spectral information of the analyzed structures. The tissue/light interaction (reflectance, absorption, scattering) generates specific spectral signatures, enabling non-invasive and non-ionizing qualitative and quantitative analysis of the biochemical composition of an object: the so-called optical biopsy. However, HSI produces a massive amount of data, and in order to extract and classify the discriminative information, machine learning (ML) techniques are required.

Given its ability to discriminate materials rapidly and over large surfaces, HSI has been previously successfully used in the field of remote sensing [4], vegetation and water resource control [5], the recycling industry [6] and forensic medicine [7]. As a result, HSI, providing a non-invasive, contrast-free optical biopsy, is able to discriminate pathological from healthy tissues [8]. For this reason, this technology has been progressively developed in the medical field over recent years [9,10] and recently found several applications in the field of gastroenterology [11].

Likewise, HSI could be easily integrated into current surgical optical navigation tools, such as laparoscopes [12], endoscopes [13] or even robotic systems; hence, it has captured the attention of the surgical community as a potential innovative surgical guidance tool [14]. In particular, HSI technology has been previously applied in digestive surgery to quantify intestinal perfusion before anastomosis during several procedures [15,16,17], as well as in the case of mesenteric ischemia [18,19], or to quantify liver perfusion [20]. A number of previous works focused successfully on the ability of HSI to discriminate between normal and tumoral tissue, particularly in prostate cancer [21], colorectal cancer [22,23,24], gastric cancer [25,26], glioblastoma [27] and head and neck cancers [28,29,30,31]. In the oncological field, advances in hyperspectral signature classification have been remarkable and led to the successful use of sophisticated deep learning algorithms [25,26,27,30,31,32]. However, these previous works were conducted ex vivo: either using surgical specimens or histopathological slides. Furthermore, only binary tissue recognition has been carried out: cancer versus the cumulative non-cancer class. Several groups have attempted to employ HSI as a surgical navigation tool for intraoperatively discriminating key anatomical structures, such as the bile duct [33], esophagus or tracheal tissue [34], parathyroid gland [35], nerves and ureter [36]. However, those previous tissue recognition works were conducted using either simple feature discrimination algorithms [33,34,35] or band selection methods [36,37].

The aim of this study was to apply HSI in combination with several advanced machine learning algorithms, particularly convolutional neural networks (CNNs) and support vector machines (SVMs), to differentiate nerves from several surrounding classes of tissues in an in vivo porcine model. These classes are artery, fat, muscle, skin and vein. We also include metallic objects (instruments) as an additional class to be recognized by the ML models.

## 2. Materials and Methods

### 2.1. Overview

In this section, we discuss the HSI image dataset, the machine learning task and training process and our performance evaluation setup. The models were trained to recognize the tissue type at any spatial location within HSI images, among a set of pre-defined tissue types. We solved this problem by training state-of-the-art models using supervised learning. This involved two stages: The first stage was the training stage, where the model parameters were automatically optimized to maximize recognition performance on a training dataset with known tissue classes provided by an expert surgeon. The second stage was the inference stage, where the predictive performance was evaluated with HSIs of subjects that are not present in the training dataset with leave-one-out cross-validation (LOPOCV). This evaluation measures the performance and generalization capability of the model.

### 2.2. Animal Characteristics

In the present study, 8 adult pigs (Large White) were included. This experiment was part of the ELIOS project (Endoscopic Luminescent Imaging for Oncology Surgery), approved by the local ethical committee on animal experimentation (ICOMETH No. 38.2016.01.085), and by the French Ministry of Superior Education and Research (MESR) (APAFIS#8721-2017013010316298-v2). All animals were managed according to French laws for animal use and care, and according to the directives of the European Community Council (2010/63/EU) and ARRIVE guidelines [38].

A 24 h preoperative fasting with free access to water was observed. Premedication was administered 10 min preoperatively, with an intramuscular injection of ketamine (20 mg/kg) and azaperone (2 mg/kg) (Stresnil, Janssen-Cilag, Beerse, Belgium). Intravenous propofol (3 mg/kg) combined with rocuronium (0.8 mg/kg) was used for induction. Anesthesia was maintained with 2% isoflurane. At the end of the procedures, pigs were sacrificed with an intravenous injection of Pentobarbital Sodium (40 mg/kg) (Exagon^®^, AXIENCE, Pantin, France), under 5% isoflurane anesthesia.

### 2.3. Surgical Procedure and Hyperspectral Data Acquisition

A midline neck incision was performed, and the neurovascular bundle of the neck was carefully dissected. Successively, the vagal nerve, common carotid artery and the common jugular vein were isolated bilaterally, and the nerves were marked using a 3-0 polypropylene suture thread. The surgical scene was stabilized using self-retaining retractors. The hyperspectral imager employed during the experiment was a commercially available push-broom scanning complementary metal–oxide–semiconductor (CMOS) compact system (TIVITA^®^, Diaspective Vision GmbH, Am Salzhaff-Pepelow, Germany) CE marked for human use, with a 640 × 476 pixel spatial resolution, acting within a spectral range from 500 to 1000 nm (5 nm spectral resolution). The camera head possessed an integrated illumination system, consisting of 6 halogen lamps. The 6 lamps illuminate the scene with incoherent white light, and the spectrometric unit of the camera analyzes the reflectance spectra. The hyperspectral camera was placed at approximately 45 cm from the surgical field, and an acquisition was taken. In order to avoid any potential bias, environmental lights were switched off, and the ventilation was paused for the few seconds required for each acquisition (<10 s). Supplementary material Figure S1 shows the RGB images of each acquisition. 

### 2.4. Imaging Postprocessing and Data Annotation

**Figure 1:**
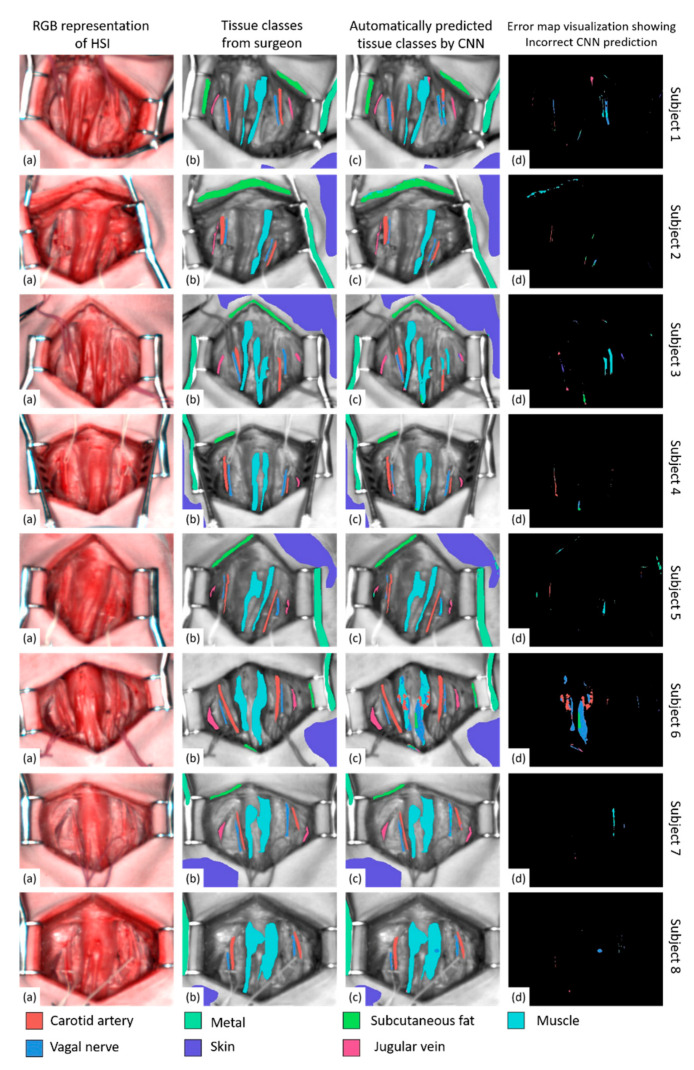
Automatic tissue recognition visualization: Visualization of automatic tissue recognition results from the CNN (convolutional neural network) model. In each row, we show images from subjects 1–8. From left to right, in columns, we show the RGB image simulated from HS (**a**), the ground truth classifications from the surgeon (**b**), the predicted classifications from the CNN (**c**) and the error map (**d**). Black pixels in the error map indicate a perfect classification (no error). Colored pixels indicate an incorrect classification, and the color provides the incorrect prediction.

Immediately after each acquisition, in the OR and with the surgical scene still available, the operating surgeon (MB), by means of an image manipulator software (GIMP, GNU Image Manipulation Program, open source), manually annotated the resulting RGB image. The annotated classes were the vagal nerve, jugular vein, carotid artery, subcutaneous fat, muscle, skin and metal of the retractor. The RGB images for each subject are shown in Figure 1a, which were provided by the camera device and synthesized from the HSI. As it is visible in the pictures, the nerves and the vessels are rather small and difficult to distinguish from the RGB images alone. For this reason, the annotation was carried out by the operating surgeon, still looking at the surgical field, in which the structures were well distinguishable, given the precise anatomical position of the dissected elements of the neck neurovascular bundle (carotid artery, jugular vein and vagal nerve). These images are visualized in grayscale with annotations overlaid as colored regions in Figure 1b. These figures are cropped and oriented similarly for better visualization. It is not feasible to correctly annotate every pixel in each image. Consequently, the annotation of each class is a subset of the pixels of the class for which the human annotator was certain of the class type. The machine learning models were trained and evaluated using only the annotated regions. One of the primary challenges is that the HSI camera has a limited resolution of 640 × 476 pixels, and, consequently, the thin structures (in particular, nerves) are challenging to recognize. Supplementary material Figures S2 and S3 show the annotations of each HSI.

### 2.5. Image Pre-Processing and Spectral Curve Distributions

The HSI images were calibrated to account for the illumination source, dark current and the current of the charge-coupled device (CCD) camera. Therefore, intensity calibration was not required as a pre-processing step. The images have 100 equally spaced wavelength bands in the range of 500 (visible green light) to 1000 nm (near-infrared). Normalization techniques are commonly applied as a pre-processing stage to reduce spectral variability caused by tissue morphology effects (changes in illumination angle, and non-flat structures) [39,40]. The most popular normalization technique was tested: standard normal variate (SNV), where each individual curve is transformed to have a zero mean and standard deviation of one. SNV renders a spectral curve invariant to linear transforms caused by, e.g., surface orientation variability. However, SNV may adversely affect recognition performance because of the potential loss of discriminative information. Models were trained by us both with and without SNV normalization to assess its benefits and limits.

**Figure 2:**
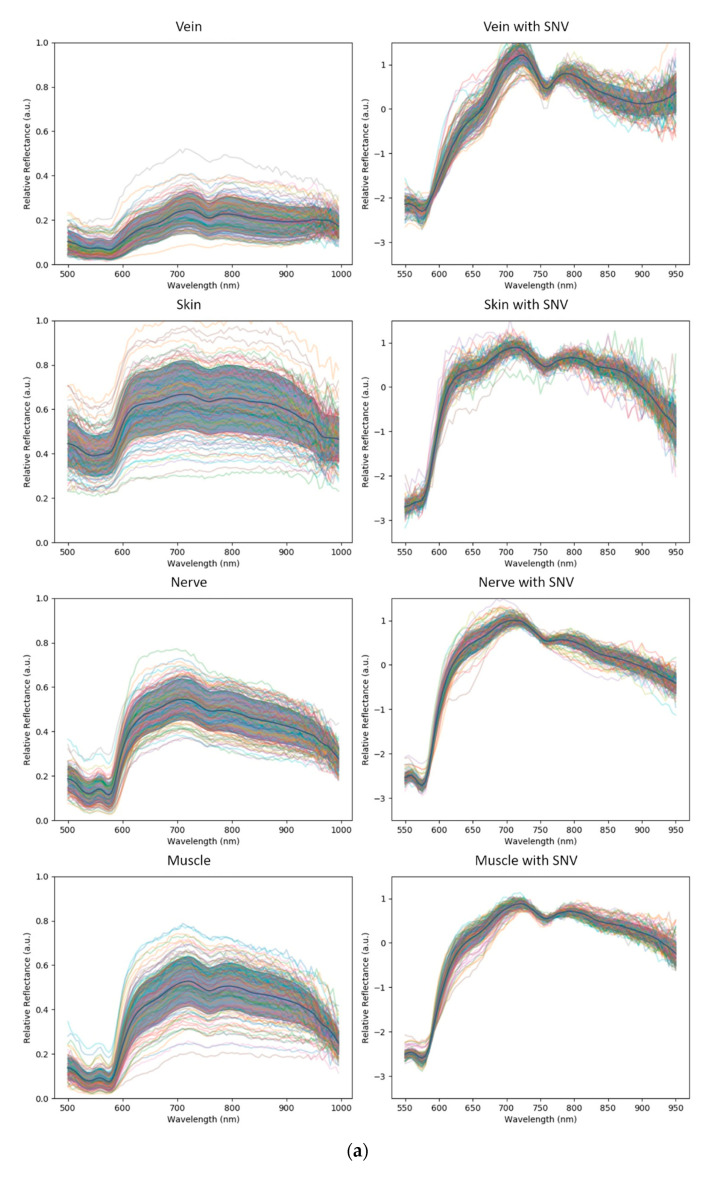

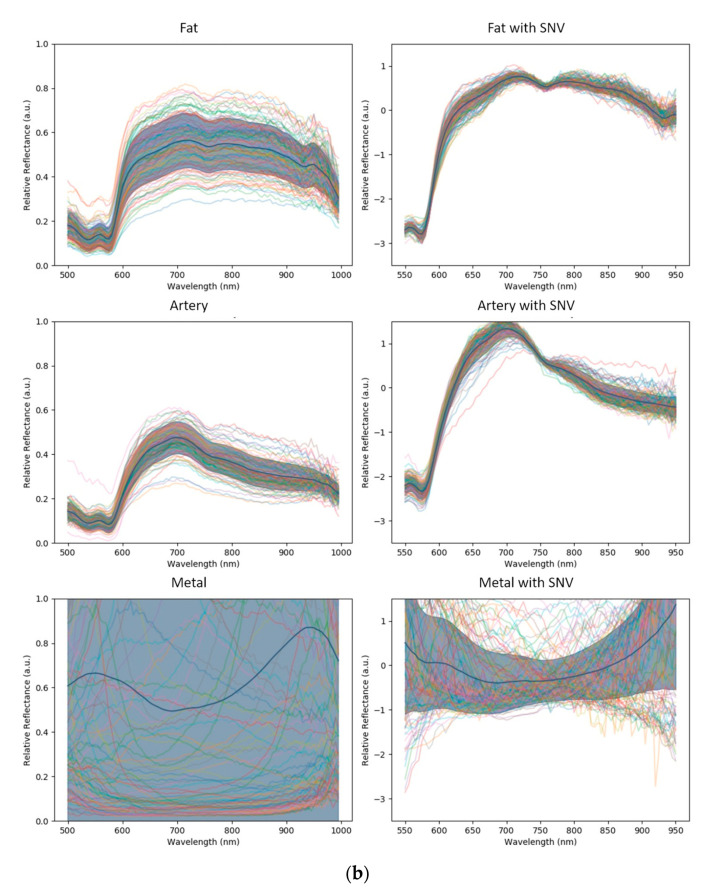
(**a**) Standard normal variate (SNV) within the classes: Spectral curve profiles of the tissue classes without and with SNV normalization. Relative absorption is plotted as a function of wavelength for each class. The mean spectral curve is shown as a black solid line, and the curve spread is shown with a gray band of one standard deviation from the mean curve. (**b**) Standard normal variate (SNV) within the classes: Spectral curve profiles of the tissue classes without and with SNV normalization. Relative absorption is plotted as a function of wavelength for each class. The mean spectral curve is shown as a black solid line, and the curve spread is shown with a gray band of one standard deviation from the mean curve. The metal class has a very large spectral distribution because of specular reflections, with a completely different profile to the tissue classes.

**Figure 3:**
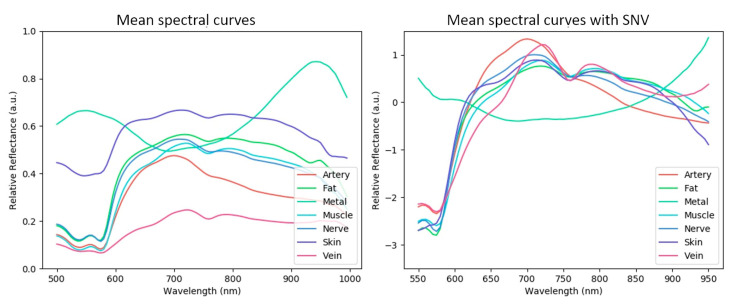
Mean spectral curves of all classes overlaid in a single plot (**left** without SNV, **right** with SNV normalization).

Before applying machine learning, a qualitative analysis of the spectral curves of each tissue class was performed to understand the intrinsic difficulty of the recognition problem. These curves are plotted in Figure 2 and Figure 3, where we show spectral curve distributions for each class with and without the use of SNV normalization. Classes that are easier to recognize by the machine have two general characteristics: (i) low intra-class variation (spectral distribution is similar for the same class) and (ii) high inter-class variation (spectral distribution is different for different classes). Intra-class variation is primarily caused by differences in the same tissue class in different subjects. In Figure 2, intra-class variation is represented by the spectral curve spread (one standard deviation from the mean, illustrated by the gray zone). Inter-class variation is visualized by differences in the mean spectral curves (solid black line). Inter-class variability is tissue-specific, where veins and skin are the most dissimilar tissues. Muscle, nerves and fat are relatively similar, indicating that recognizing them with machine learning is not trivial. The metal class has a strongly different profile compared to the tissue classes. The large intra-class variation of metal can be explained by strong specular reflections.

It is clear that intra-class variability was reduced by SNV normalization, but inter-class variability was also reduced. The mean spectral curves of all classes are plotted together in Figure 3, showing the curves without and with SNV normalization (left and right graphs of Figure 3, respectively).

### 2.6. Machine Learning Recognition Problem

**Figure 4:**
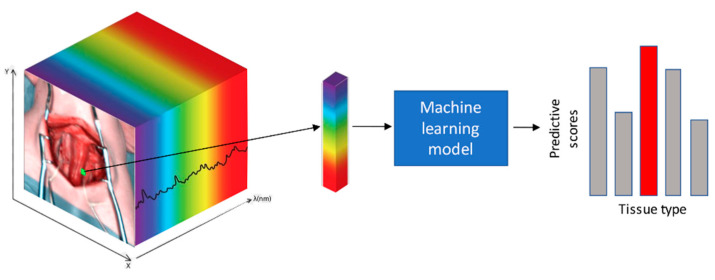
Schematic representation of hyperspectral (HS)-based tissue class recognition: Illustration of the general tissue recognition problem with HS image data. The core task is to recognize the tissue type at a given spatial location within an HS image. A sub-volume is extracted at the spatial location with a small spatial window, which is passed to the predictive machine learning model. Predictive scores are then automatically generated for each tissue class. Finally, the class with the highest predictive score is selected.

Given a hyperspectral image, for each spatial coordinate (pixel), a sub-volume centered on the pixel is extracted, which is then input to a predictive machine learning model. This model produces a score for each tissue class, where a higher score indicates a greater likelihood of the tissue class being present at the spatial coordinate. Finally, the pixel is assigned to the class with the highest predictive score. This process repeats for each pixel of interest. This machine learning recognition problem is illustrated in Figure 4.

### 2.7. Machine Learning Models

Various machine learning models have been applied to other HSI image classification problems including medical imaging and remote sensing, and there is no single model that performs best on all datasets [41,42]. Consequently, the two most successful models for HSI image segmentation were studied in this work: support vector machines (SVMs) [41,43,44] and convolutional neural networks (CNNs) [42,45].

### 2.8. Support Vector Machine (SVM)

SVMs have been shown to work well for HSI classification problems, particularly remote sensing [46,47,48]. They are simple to train and they offer good performance when the training set size is limited [41] and when the input data are high-dimensional, as is the case for our dataset. An SVM classifier fits a decision boundary (hyperplane) in a multidimensional feature space. The feature space is constructed by transforming the data points using a kernel, such as radial basis functions, to the feature space. Training is then performed to determine a hyperplane that separates the classes in the feature space. Once trained, a new spectral curve at a given pixel is first transformed to the feature space and then classified using the hyperplane. SVMs generalize naturally to multi-class classification problems such as ours, and they have been shown to outperform other classical machine learning models in HSI classification problems such as k-nearest neighbor (KNN) and decision trees [49]. We constructed the feature space using the relative absorption of each wavelength in the HSI sub-volume centered at a given pixel. Consequently, the feature space had 100 dimensions (one dimension per wavelength).

### 2.9. Convolutional Neural Network (CNN)

The second model is a CNN, shown to work well with many types of image data including HS [42,45,50,51]. A CNN is a special type of feed-forward neural network where neurons are arranged in a series of hidden layers. The image is fed into the first layer, and the purpose of the layer is to extract meaningful features to aid classification. These features are fed into the second layer, where higher-level features are computed from the inputted features, and the process repeats until the last layer. Finally, a classification decision is made using the features at the last layer. CNNs are specially designed so that the features are extracted using convolutional filters. The filter weights are trainable parameters of the mode, and during learning, they automatically adapt to extract features that are most pertinent to the specific classification task.

### 2.10. Implementation and Training

For each HS image in the dataset, we sampled the sub-volume centered on every annotated pixel. Spatial patches of 5 × 5 pixels were extracted to form 5 × 5 × 100 sub-volumes. The third dimension corresponds to the wavelength dimension with the 100 bands. The dataset was then composed of 213,203 samples.

Those sub-volumes were successively split into training and testing sets respecting the LOPOCV process to train and evaluate the performance of the model. LOPOCV is a standard practice in medical image classification with small datasets, and it ensures that a model is not trained and tested on data from the same subject. Performance statistics with LOPOCV measure the generalization performance of a classifier on new subjects. LOPOCV was performed by partitioning the subjects into 8 subsets, S1, S2 … S8, where each subset S*i* was formed of every sub-volume extracted from all subject HS images excluding the *i*th subject. The models were trained on a subset S*i*, and then performance was tested on the *i*th subject HS image. The process was repeated 8 times for each subject.

**Figure 5:**
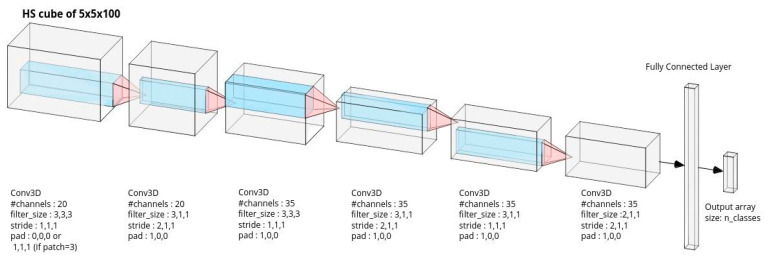
Convolutional neural network architecture: The input is an HSI sub-volume centered on a given pixel, with 5 × 5 spatial dimensions and 100 wavelength dimensions. Down-sampling convolutional operations are applied to transform the input to a final 1D feature space vector, which is followed by a final fully connected layer to produce the tissue prediction scores.

The SVM classifier was implemented with Python’s scikit-learn library [52]. Auto-scaling was applied to each spectral band (unit variance and mean centering) [53] to eliminate the influence of variable dimensions. Default SVM parameters were used from Python’s sklearn.svm.SVC class (radial basis kernels (RBFs of degree 3 and regularization parameter C=1). The CNN was implemented in Pytorch v1.2 using an established 3D CNN neural network architecture [50] that has been implemented in a public code. The CNN processed a sub-volume centered around a given pixel using a 5 × 5 spatial window, and it had 32,628 trainable parameters with 7 hidden layers. The first 6 layers were convolutional, and the last hidden layer was fully connected. The final output layer had 7 neurons, where 7 is the number of classes (6 tissue classes and the metal class). Each output layer computed the predictive score of each class. Finally, the class with the highest score was selected. The architecture of the CNN is provided in Figure 5, for full reproducibility. The CNN was trained using Pytorch’s backpropagation implementation with Adam optimization (0.001) [54]. As the dataset was highly imbalanced with some classes significantly more represented than others, training was implemented with a weighted cross-entropy loss with weight inversely proportional to the number of training samples per class. This is standard practice to prevent the classifier attempting to maximize performance for the most represented class (in our case, the skin class).

Training was performed on a DGX V1 (Nvidia corp.) equivalent deep learning server (Cysco inc.), taking approximately 15 h for the CNN and 25 h for the SVM. Training was performed 8 times in order to implement LOPOCV. Each training session had seven training images and one test image, and training was repeated eight times so that every image was used once as a test image.

### 2.11. Performance Metrics and Statistical Methods

Performance was evaluated with standard metrics used in machine learning, implemented by Python scikit-learn (version 0.23, https://scikit-learn.org; (accessed on 2 January 2020)). Multi-class Type 1 and Type 2 errors were evaluated and are presented as confusion matrices. The area under the receiver operator curve (AU-ROC), sensitivity, specificity and Sørensen–Dice coefficient score (DCS) were used to evaluate class-specific performance using ‘one versus all’ and macro averaging. DCS is commonly used to evaluate the classification accuracy of machine learning models at the pixel level in the medical imaging community, and it is equivalent to the F1 score and represents the harmonic mean of a classifier’s positive predictive value and its sensitivity. Statistically significant differences were computed with a two-tailed paired *t*-test (α = 0.05) similarly to previous works in HSI classification analysis [55] using Excel (Microsoft Office 365, Microsoft, Redmond, WA, USA).

## 3. Results

### 3.1. Model Configurations

We compare the performance of different models to measure performance across two axes. The first axis is the model type (SVM with radial basis kernel versus CNN), and the second is with and without standard normal variate (SNV) normalization. This produces four combinations, named as follows:

1. CNN: CNN trained without SNV normalization;
2. CNN+SNV: CNN trained with SNV normalization;
3. SVM: SVM trained without SNV normalization;
4. SVM+SNV: SVM trained with SNV normalization.

### 3.2. Confusion Matrices

**Figure 6:**
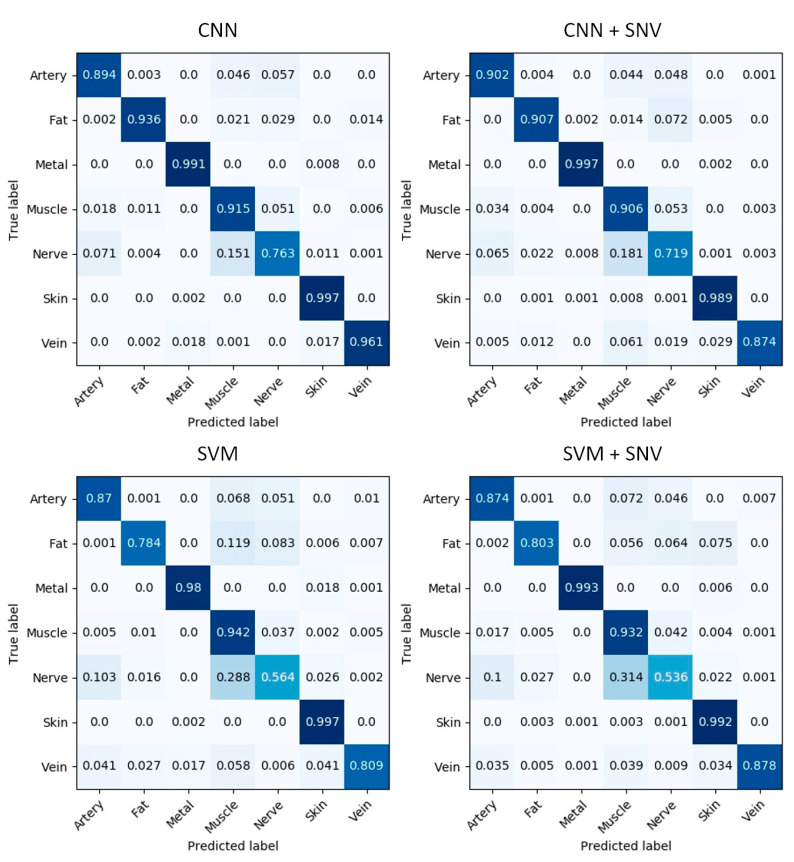
Confusion matrices: predictive performance of 4 different models visualized as confusion matrices.

The confusion matrices provide a compact representation of the multi-class classification performance in Figure 6. One confusion matrix is presented per model, and an item (i,j) in each matrix shows the proportion of pixels of class i that are predicted to be of class j. The confusion matrices are normalized such that each row adds up to 1. Consequently, the diagonal entries provide the true positive rate (TPR) of each class, equivalently, its sensitivity. The off-diagonal entries (i,j) with j>i provide the false positive rate (FPR, Type 1 error) for class i with respect to class j. The off-diagonal entries (i,j) with i>j provide the false negative rate (FNR, Type 2 error) for class i with respect to class j. A confusion matrix of a perfectly performing model has zeros everywhere, except for ones along the main diagonal.

The CNN model achieves a sensitivity of 89.4% and above for all tissue classes except for nerve, which has a sensitivity of 76.3%. Skin tissue has the lowest class confusion among all tissue classes with a sensitivity of 99.7%. Considering the CNN versus the SVM model, the CNN achieves a much higher sensitivity for fat (93.6% versus 78.4%), nerve (76.3% versus 56.4%) and vein (96.1% versus 80.9%) tissues. The remaining classes (artery, skin, muscle and metal) have similar sensitivities within 3% of each other for either model. These results indicate that SNV normalization, in general, harmed the performance of the CNN. Instead of improving performance thanks to a reduction in intra-class variability (for which SNV normalization is designed), it harmed the performance of the CNN because it filtered out useful information that can be exploited by the CNN via deep learning. One can see that this lost information is useful to the CNN to obtain a good classification performance, especially for nerve and vein tissues. Consequently, the CNN model learned useful spatio-spectral features via deep learning that are able to handle inter-class variability without SNV normalization. In contrast, the benefit of SNV normalization on the SVM model is mixed. SNV normalization improves the sensitivity of the vein class with the SVM model (80.9% versus 87.8%), but for the other classes, the sensitivity is similar and within 2% for either model. Therefore, the SVM, which does not learn deep features, can sometimes achieve better results by reducing intra-class variation with SNV normalization, but there is not a systematic improvement for all tissue classes.

### 3.3. Performance Visualizations

The outputs of the best model (CNN) are visualized in Figure 1. This figure is arranged into rows and columns as follows. Each row corresponds to a different subject, and there are four images per row. From left to right, these are as follows: (a) the RGB simulated from the HSI image, (b) the corresponding ground truth tissue annotations provided by the surgeon (overlaid on grayscale images for better visibility), (c) the predicted tissue annotations computed by the model and (d) an error map visualization. The error map shows where predictions from the model deviated from the ground truth annotations. Pixels in the error map that are black indicate a perfect agreement between the predicted and ground truth tissue classes. Colored pixels in the error map indicate a prediction error, and a color is provided for the class that was incorrectly predicted. A perfectly performing model has a completely black error map. When a mistake is made by the model at a spatial location, we visualize the incorrectly predicted class.

For all subjects, we clearly see excellent recognition performance for the skin, subcutaneous fat, carotid artery, jugular vein and metal classes, with very low errors. For the muscle and vagal nerve classes, we observe a strong performance for subjects 1, 2, 4, 5, 7 and 8, with few errors (indicated by mostly black pixels in the error visualization maps). There are two main sources of error: in subject 3, nerve was mistaken for muscle, and in subject 6, muscle was mistaken for nerve and artery.

### 3.4. Further Quantitative Analysis and Statistics

**Figure 7:**
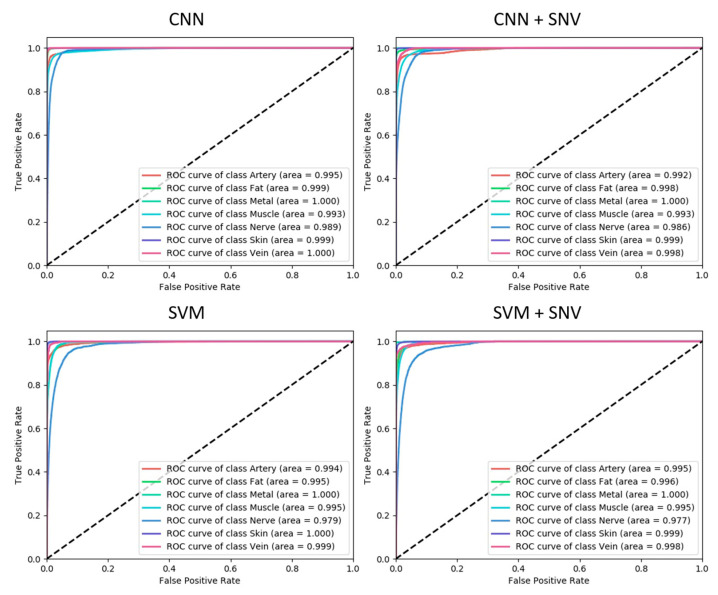
Receiver operator characteristic curves: Receiver operator characteristic curves for the four models. In each figure, we plot the ROC curves for one model for all classes.

Model performance was further analyzed with receiver operator characteristic (ROC) curves and other performance metrics: Dice similarity coefficient (DSC), sensitivity and specificity. An ROC curve measures the performance of a classifier in terms of false positive and true positive rates. These are generated for each model and for each tissue class as follows. For each class, the prediction scores for all pixels in the test images are computed, and then the false positive and true positive rate is computed using a varying detection threshold. For a lower threshold, a higher false positive rate is obtained, but at the cost of a lower true positive rate. The ROC curve is generated by sweeping the detection thresholds from low (true positive rate of zero) to high (true positive rate of one). ROC curves are plotted for the four models in Figure 7. A high recognition performance is indicated by a high area under the curve (ROC-AUC), and the ROC-AUC summarizes the balance between false positives and true positives, and it is also insensitive to high class imbalance, which is the case with this dataset where some classes, e.g., the skin class, are significantly more represented than others, e.g., the nerve class. The ROC-AUC is also equivalent to the probability that a randomly selected sample will have a higher classification score for its true class compared to other classes. For the CNN model, all ROC-AUCs are above 0.99, which is considered an outstanding performance [56]. For the other models, ROC-AUCs are similar, but the ROC-AUC for the nerve class with the SVM and SVM+SNV models is consistently lower compared to CNN and CNN+SVM.

**Figure 8:**
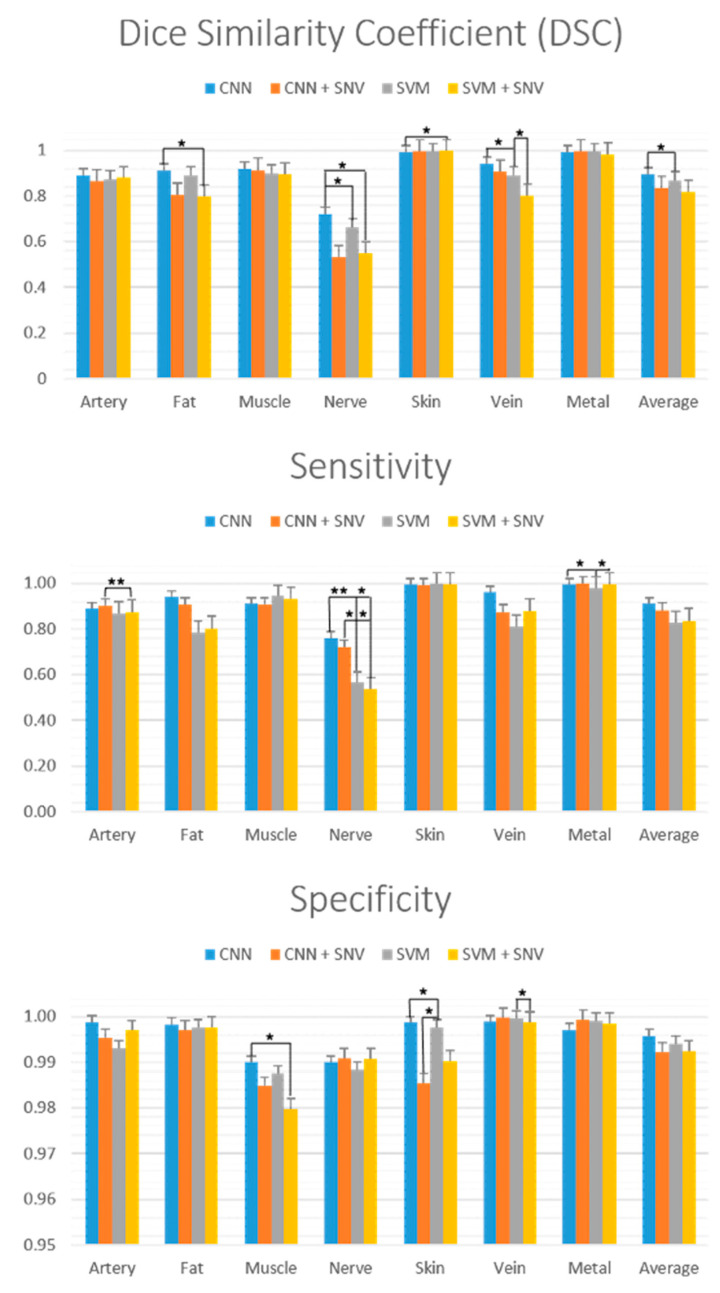
Performance of the four classification models in terms of Dice similarity coefficient (DSC) (**top**), sensitivity (**middle**) and specificity (**bottom**). Error bars represent 1 standard error. Brackets with * and ** denote statistical significance at *p* < 0.05 and <0.01 respectively.

The DCS (also called the Dice coefficient or F1 score), sensitivity and specificity performances of the four models are plotted in Figure 8. These are commonly used performance metrics used in the medical image processing literature. The DCS provides the harmonic mean of a classifier’s precision and sensitivity [57]. Solid bars represent the average performance metric for each model and for each class, computed by averaging the performance metric across each of the 8 images. Error bars represent one standard error. Statistically significant differences were computed with a two-tailed paired *t*-test (α = 0.05) similarly to previous works in HSI classification analysis [55]. Significance is indicated by the bracketed bars in Figure 8, with one star for *p* < 0.05 and two stars for *p* < 0.01. Sensitivity and specificity scores are additionally summarized in Table 1. Regarding the DSC, the CNN model generally has a better average DSC compared to CNN+SNV, but the difference is not statistically significant. In contrast, the CNN model obtains a significantly better DSC performance compared to SNV for the nerve (*p* = 0.010) and vein (*p* = 0.044) classes. No other models (CNN+SNV, SVM, SVM+SNV) obtain a significantly better DSC compared to the CNN model. Concerning sensitivity, there are no significant differences between the CNN and CNN+SNV models. In contrast, the CNN model has a significantly better sensitivity compared with the CNN and CNN+SNV models for the nerve class (*p* = 0.0038 and *p* = 0.0011, respectively). Concerning specificity, there is no significant difference between the CNN and CNN+SNV models. The CNN model has a significantly better specificity compared to the SVM model for skin tissue (*p* = 0.021) and a better specificity compared to the SVM+SNV model for muscle tissue (*p* = 0.036). The DSC, sensitivity and specificity were averaged across all tissue classes, shown in the right-most bars in Figure 4. The CNN model obtains the highest average scores for all three metrics.

## 4. Discussion

In the current work, despite the relatively small sample size (eight pigs), we were able to recognize six different tissue classes with a high degree of accuracy using HSI in combination with advanced machine learning algorithms in an in vivo porcine model. The CNN proved to be the best performing model. There are three key advantages of CNNs that make them very popular for image classification: (1) They automatically learn a hierarchical representation of the data tailored to the specific classification task at hand. Unlike classical methods such as SVMs or decision trees, the CNN learns the best features from the raw image data. This eliminates tedious feature engineering tasks. (2) CNNs are general purpose and highly flexible, and model complexity can be modified with different CNN designs. (3) CNNs can be trained efficiently using highly parallelized implementations such as Pytorch [58]. The main disadvantage of CNNs is that they typically have many parameters to learn (several millions is not uncommon), and this can make them susceptible to poor performance if training data are limited. Various techniques exist to overcome this, including using smaller models, simulating additional training data (data augmentation) and training by randomly deactivating neurons (drop-out) or by reusing parameters from a CNN trained on a related task with a greater amount of data (transfer learning). The CNN model used in this study is relatively small with 32,628 parameters, and we were able to train well-performing models that, in general, outperformed the SVM models. Consequently, it is possible to solve multi-class tissue classification with HSI using a relatively small CNN, and therefore to benefit from automatic discovery of relevant spatio-spectral features within the HSI data using deep learning.

Hyperspectral imaging is a powerful tool that enables unraveling the invisible in an objective way, relying on curves obtained by the interaction of the emitted light with the biochemical tissue components. Given the contrast-free and non-invasive nature of this versatile technology, it has the potential of becoming a future intraoperative guidance navigation tool. As a result, the rationale behind our experimental study was to test the reliability of HSI in recognizing multiple tissue classes in vivo, in order to develop, at a later stage, an HSI-based automatic intraoperative navigation system for use in the clinical setting.

More than a decade ago, in a pioneer work, Zuzak et al. successfully differentiated the portal vein from the biliary duct and the hepatic artery, by means of a laparoscopic hyperspectral imager prototype [33]. In their experimental proof of the concept, the spectral curves of the different tissue types were analyzed using PCA (principal component analysis). However, despite the promising results, the long acquisition time of the HSI laparoscopic prototype and the scarce reproducibility of the PCA-based tissue discrimination impaired the clinical translation of this technology.

Recently, other authors attempted to discriminate anatomical structures by means of HSI intraoperatively [36,37]. However, in both works, the authors did not rely on the spectral curves for the discriminative analysis, rather attempting to select the best spectral bands in order to obtain an RGB enhanced image, in order to highlight, for the human eye, the chosen target structures, such as nerves or ureters. This approach has the potential of allowing for a rapid analysis, once the best band is selected. However, this principle very much resembles the concept of NBI (narrow band image), a band selection modality, largely utilized in diagnostic gastrointestinal endoscopy. In fact, the light emitted in this particular band (415 nm) is able to delineate the surface micro-vessels, which are, due to neoplastic neoangiogenesis, more represented within cancerous lesions, allowing for discriminating normal from cancerous or pre-cancerous mucosa. Despite the remarkable results of both groups, band selection only partially uses the potential of HSI and still requires a substantial interpretation of the operator to discriminate the enhanced structure. The utilization of advanced machine learning algorithms to reliably detect the slight differences within spectral curves of different tissues enormously reduces the human interpretation bias, heading towards precision surgery.

Enormous advances have been made in this sense, in the field of HSI-based cancer detection. In particular, deep learning has been successfully coupled to HSI to detect, with high accuracy, gastric cancer from healthy tissue on fresh samples [26] or microscopic slides [25]. Recently, in a remarkable work involving 82 patients, a group could identify salivary gland and thyroid tumors on operative specimens, with impressive precision [32]. Additionally, the authors were able to demonstrate the superiority of HSI in detecting the specific cancer types against fluorescence dye-based imaging techniques. All these innovative works have the merit of discriminating cancerous lesions with a high degree of accuracy by coupling HSI to deep learning algorithms. However, in contrast to those previous works, in which only cancer has to be recognized over the non-cancer merged classes, in our setup, we were able to discriminate precisely among six different tissue classes in an in vivo large animal model.

Since we had a limited amount of data, we decided to use all data for the training phase, and by employing the leave-one-patient-out cross-validation (LOPOCV) technique, we could reliably verify our algorithms’ performance. On the other hand, a cross-validation in which only a subset of data is used as training and the rest as testing data, as performed in a number of previous works, has the disadvantage of being less reliable than the LOPOCV method.

During our experimental setup, the ground truth was provided by annotations made intraoperatively by a fully trained surgeon. As explained in the methods, the annotations were performed in the OR, having the surgical scene still available and magnifying the RGB images associated with each hyperspectral image on the computer’s screen. In fact, annotation based on the RGB images alone in a subsequent postprocessing phase would have certainly led to annotation mistakes. However, only the tissue classes that, after accurate dissection, could be visually recognized with absolute confidence were annotated, and this was always the case of the elements of the neurovascular bundle (carotid artery, jugular vein and vagal nerve), given their defined anatomical position. As a consequence, a large part of the image remained unlabeled, since without systematical histopathological mapping (which is hardly practicable in reality), it is not possible to visually discriminate certain tissue types, such as fat from connective tissue, which might be encountered over large areas.

Despite the limited sample size, we were able to achieve an excellent discrimination accuracy. This result supports the fitness of CNNs for objectively interpreting HSI datasets.

Previously tissue discrimination was achieved by qualitative analysis of the spectra obtained by means of the probe-based diffuse reflectance spectroscopy (DRS) [59]. As noted in our findings, those authors also observed analogous patterns within curves of fatty tissue, muscle and nerves, and they were able to find discriminative characteristics for these tissue types only by using an additional DRS sensor able to analyze the spectrum beyond 1000 nm. However, the hyperspectral imager we employed reached a maximal spectral range at 1000 nm, but still, we were able to successfully discriminate those ambiguous tissue types. This was achieved with the fine adjustment of the CNN parameters, demonstrating that CNNs are particularly suitable to analyze, with high precision, large hypercubes.

However, our work has several limitations. Firstly, the HSI system we employed has a limited spectral range (500–1000 nm), a relatively low spectral resolution (5 nm) and a relatively low spatial resolution (640 × 476 pixel). This could have negatively affected our results. However, we employed this system since it is approved for human use, and fitting recognition algorithms on such a system would enable a faster clinical translation of our research. Secondly, the limited sample size represents a considerable limitation of this study. Thirdly, among all tissue types, nerves displayed the lowest sensitivity and specificity. This could result from the fact that they are the tiniest structures compared to all others, and for this reason, they are underrepresented in our setting. Another reason could be that the underrepresented nerves displayed similar spectral curves to the overrepresented muscles, and this contributed to confusing the algorithms.

In conclusion, this work demonstrates that an in vivo recognition of anatomical key structures (in particular, blood vessels and nerves) using hyperspectral technology in association with deep learning is possible and highly accurate. Future efforts will be allocated to developing an advanced HSI-based navigation tool to support the mental anatomical map of the surgeon, as a tool to avoid inadvertent injuries to key anatomical structures. Furthermore, as the era of autonomous robotic surgery emerges, automatic tissue classification will be an essential tool to facilitate safe autonomous procedures, and HSI combined with deep learning may prove a key enabling technology. As future follow-up work, we aim to enlarge the study with more subjects and to repeat the experimental protocol with human data captured in an observational clinical trial.

## Figures and Tables

**Table 1:** Performance of the four classification models in terms of sensitivity (**top**) and specificity (**bottom**).

**Sensitivity**	**Artery**	**Fat**	**Metal**	**Muscle**	**Nerve**	**Skin**	**Vein**
CNN	0.894 ± 0.172	0.936 ± 0.102	0.991 ± 0.016	0.915 ± 0.183	0.763 ± 0.203	0.997 ± 0.004	0.961 ± 0.067
CNN + SNV	0.902 ± 0.145	0.907 ± 0.213	0.997 ± 0.002	0.906 ± 0.211	0.719 ± 0.218	0.989 ± 0.014	0.874 ± 0.267
SVM	0.870 ± 0.132	0.784 ± 0.274	0.980 ± 0.022	0.942 ± 0.119	0.564 ± 0.262	0.997 ± 0.002	0.809 ± 0.255
SVM + SNV	0.874 ± 0.132	0.803 ± 0.216	0.993 ± 0.008	0.932 ± 0.140	0.536 ± 0.261	0.992 ± 0.008	0.878 ± 0.190
**Specificity**	**Artery**	**Fat**	**Metal**	**Muscle**	**Nerve**	**Skin**	**Vein**
CNN	0.996 ± 0.008	0.998 ± 0.002	0.998 ± 0.003	0.994 ± 0.005	0.990 ± 0.185	0.997 ± 0.008	0.999 ± 0.001
CNN + SNV	0.993 ± 0.140	0.998 ± 0.001	0.999 ± 0.001	0.988 ± 0.137	0.988 ± 0.020	0.998 ± 0.005	1.000 ± 0.001
SVM	0.997 ± 0.003	0.998 ± 0.002	0.999 ± 0.002	0.980 ± 0.022	0.991 ± 0.015	0.990 ± 0.013	0.999 ± 0.001
SVM + SNV	0.995 ± 0.006	0.997 ± 0.003	0.999 ± 0.001	0.985 ± 0.012	0.991 ± 0.016	0.985 ± 0.024	1.000 ± 0.000

## Data Availability

All data will be made available upon reasonable request to the corresponding author.

## Supplementary Materials

The following are available online at https://www.mdpi.com/article/10.3390/diagnostics11081508/s1, Figure S1: RGB images of the dataset (uncropped), Figure S2: dataset annotation for subjects1-4, Figure S3: dataset annotation for subjects 5–8.
