# Supplementary material for: Deep Learning Analysis of In Vivo Hyperspectral Images for Automated Intraoperative Nerve Detection
Source: Diagnostics (Basel). 2021 Aug 21;11(8):1508. doi: 10.3390/diagnostics11081508 (PMC8391550; doi:10.3390/diagnostics11081508)
Supplement: Supplementary file 1 [file diagnostics-11-01508-s001.zip › diagnostics-1304288-supplementary.pdf]

Supplementary material

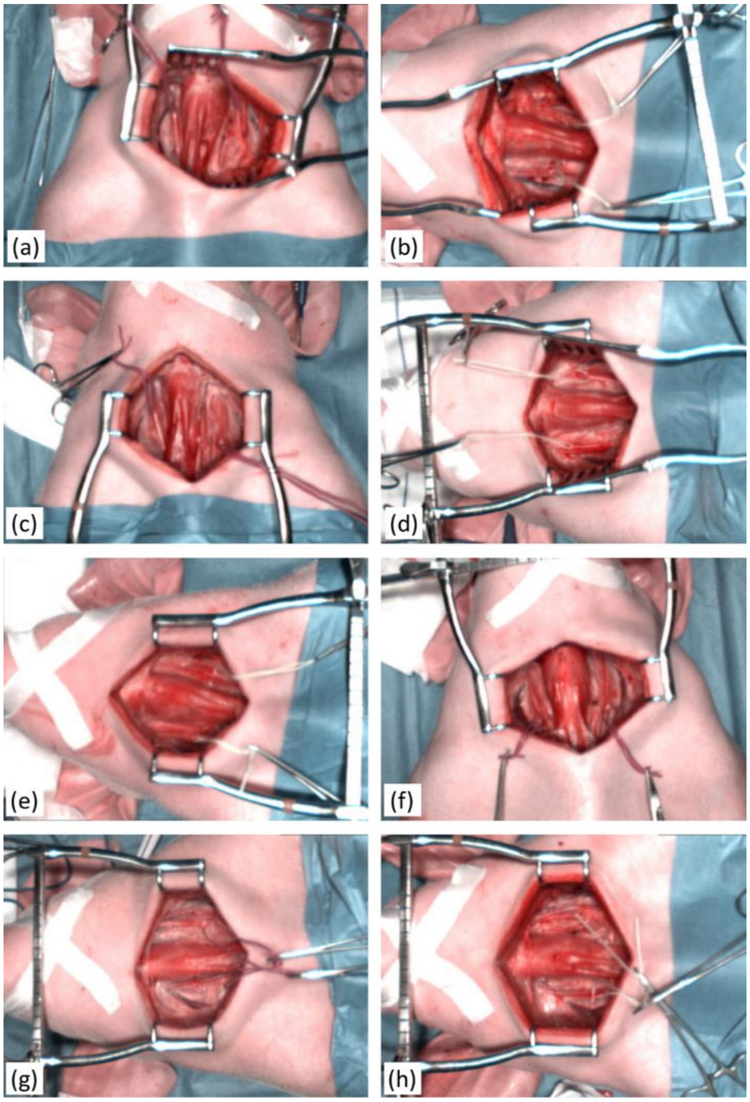

Figure S1. Dataset as the surgeon sees it***:*** Illustration of the dataset. Eight RGB images of the exposed neck are shown with one image per subject. Each image has been synthesized from an HS image (640 x 476 px).

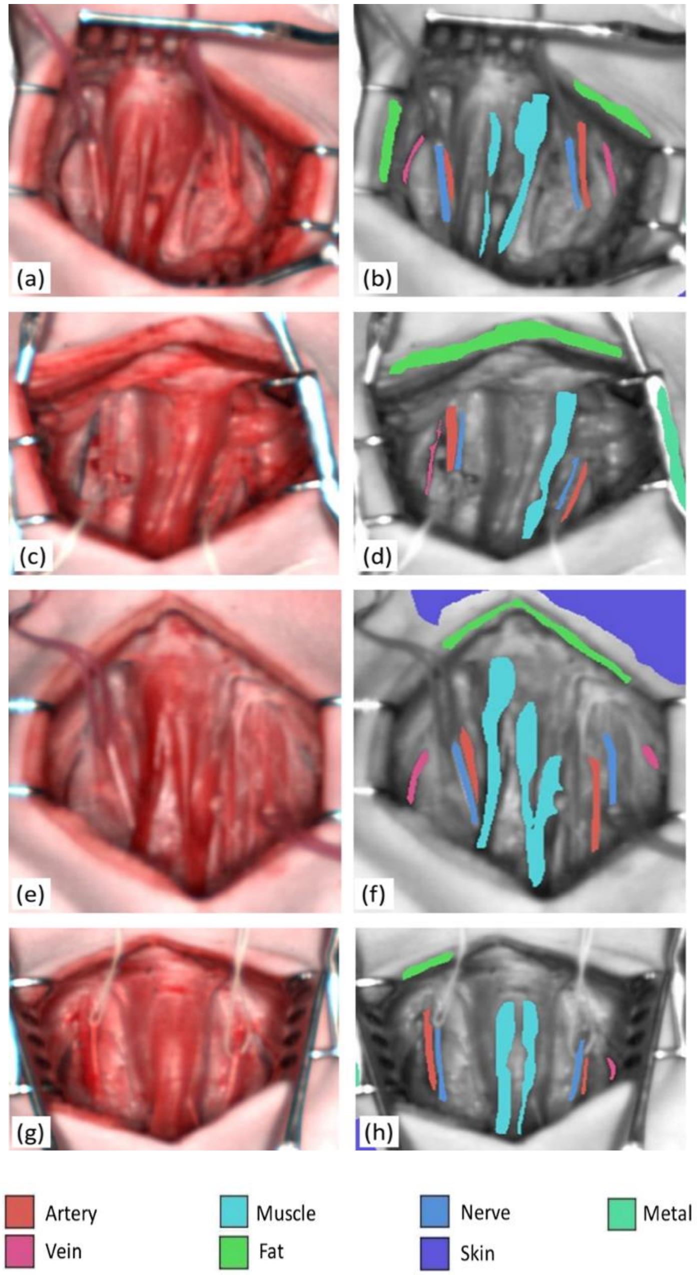

Figure S2. Dataset Annotation A: Four of the RGB images (subjects 1 – 4) cropped to the exposed region, shown in the left column. The corresponding color-coded annotations are shown in the right column, overlaid over the image in grayscale. Low image quality and blur is normal because the images are simulated from HSI with resolution 640 x 476 px.

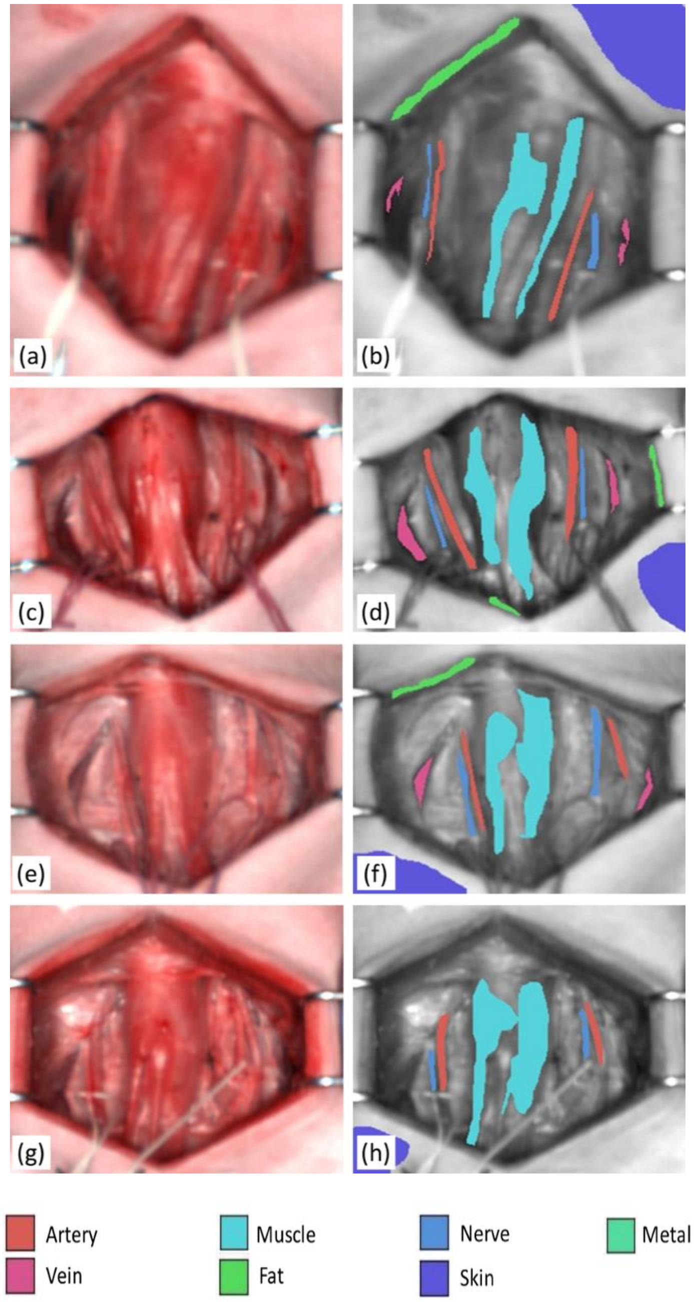

Figure S3. Dataset Annotation B: Four of the RGB images (subjects 5 – 8) cropped to the exposed region, shown in the left column. The corresponding color-coded annotations are shown in the right column, overlaid over the image in grayscale. Low image quality and blur is normal because the images are simulated from HSI with resolution 640 x 476 px.
